# Gait-Based AI Models for Detecting Sarcopenia and Cognitive Decline Using Sensor Fusion

**DOI:** 10.3390/diagnostics14242886

**Published:** 2024-12-22

**Authors:** Rocío Aznar-Gimeno, Jose Luis Perez-Lasierra, Pablo Pérez-Lázaro, Irene Bosque-López, Marina Azpíroz-Puente, Pilar Salvo-Ibáñez, Martin Morita-Hernandez, Ana Caren Hernández-Ruiz, Antonio Gómez-Bernal, María de la Vega Rodrigalvarez-Chamarro, José-Víctor Alfaro-Santafé, Rafael del Hoyo-Alonso, Javier Alfaro-Santafé

**Affiliations:** 1 Department of Big Data and Cognitive Systems, Instituto Tecnológico de Aragón (ITA), María de Luna 7-8, 50018 Zaragoza, Spain; raznar@ita.es (R.A.-G.); plazaro@ita.es (P.P.-L.); ilopez@ita.es (I.B.-L.); psalvo@ita.es (P.S.-I.); ahernandez@ita.es (A.C.H.-R.); vrodrigalvarez@ita.es (M.d.l.V.R.-C.); rdelhoyo@ita.es (R.d.H.-A.); 2 Podoactiva Research & Development Department, Biomechanical Unit, Parque Tecnológico Walqa, Ctra. N330a Km 566, 22197 Cuarte, Spain; marinaazpiroz@podoactiva.com (M.A.-P.); martinmorita@podoactiva.com (M.M.-H.); antoniogomez@podoactiva.com (A.G.-B.); victoralfaro@podoactiva.com (J.-V.A.-S.); javieralfaro@podoactiva.com (J.A.-S.); 3 Facultad de Ciencias de la Salud, Universidad San Jorge, Villanueva de Gállego, 50830 Zaragoza, Spain; 4 Department of Podiatry, Faculty of Health Sciences, Manresa University, 08243 Manresa, Spain

**Keywords:** inertial measurement unit, wearable sensor, artificial intelligence, machine learning, human pose estimation, musculoskeletal disorders, older adults

## Abstract

**Background/Objectives**: Sarcopenia and cognitive decline (CD) are prevalent in aging populations, impacting functionality and quality of life. The early detection of these diseases is challenging, often relying on in-person screening, which is difficult to implement regularly. This study aims to develop artificial intelligence algorithms based on gait analysis, integrating sensor and computer vision (CV) data, to detect sarcopenia and CD. **Methods**: A cross-sectional case-control study was conducted involving 42 individuals aged 60 years or older. Participants were classified as having sarcopenia if they met the criteria established by the European Working Group on Sarcopenia in Older People and as having CD if their score in the Mini-Mental State Examination was ≤24 points. Gait patterns were assessed at usual walking speeds using sensors attached to the feet and lumbar region, and CV data were captured using a camera. Several key variables related to gait dynamics were extracted. Finally, machine learning models were developed using these variables to predict sarcopenia and CD. **Results**: Models based on sensor data, CV data, and a combination of both technologies achieved high predictive accuracy, particularly for CD. The best model for CD achieved an F1-score of 0.914, with a 95% sensitivity and 92% specificity. The combined technologies model for sarcopenia also demonstrated high performance, yielding an F1-score of 0.748 with a 100% sensitivity and 83% specificity. **Conclusions**: The study demonstrates that gait analysis through sensor and CV fusion can effectively screen for sarcopenia and CD. The multimodal approach enhances model accuracy, potentially supporting early disease detection and intervention in home settings.

## 1. Introduction

As a population ages, the likelihood of developing various diseases increases [1]. Among these, degenerative musculoskeletal and cognitive diseases are highly prevalent [1], with sarcopenia and cognitive decline (CD) being among the most common conditions in individuals over 60 years of age [2,3].

Despite their high prevalence, these diseases are often detected by healthcare professionals only after they have progressed to advanced stages [4]. This delay is due, among other factors, to the limited diagnostic support tools available to healthcare providers, which primarily rely on in-person screening questionnaires [4,5,6,7]. These tools are often difficult to implement in routine clinical practice for various reasons [4]. As a result, patients experience poorer health outcomes and a reduced quality of life [8,9], which, in many cases, becomes irreversible [10]. Additionally, this situation imposes a greater burden and increased costs on both the healthcare system and patients’ families [11,12].

In response to this situation, various screening tools and systems have been developed [13,14,15]. Many of these are based on gait analysis, which serves as a potential indicator of overall health status and functionality and is associated with the presence of various diseases and age-related conditions [16,17,18,19].

In particular, machine learning (ML) techniques, such as those explored in this study, address the previously mentioned challenges. Specifically, continuous monitoring through sensors can enable automatic screening for diseases like sarcopenia and CD, providing earlier detection and reducing the reliance on in-person assessments, without requiring healthcare professionals’ intervention until the point of final diagnosis. Compared to traditional diagnostic tools, ML models demonstrate improved accuracy and efficiency, potentially alleviating the strain on healthcare systems while delivering better outcomes for patients. The main idea of this study is to lay the groundwork for developing an automatic screening system using ML models that analyze gait behavior, enabling healthcare professionals to confirm positive cases (sarcopenia or CD) with appropriate diagnostic tests.

### 1.1. Related Work

Regarding related work on the detection of these diseases, most research efforts have focused on developing screening systems using inertial measurement units (IMUs) [13,20], which, combined with artificial intelligence (AI), could enable a continuous and automated home screening method [13,14]. However, most studies have focused on detecting a single disease, either sarcopenia [21,22,23,24,25,26] or CD [27,28,29].

Regarding the placement of IMUs, state-of-the-art studies typically focus on fixed positions on the feet [21,22,23,24], hips, or lumbar area [25]. These areas are considered ideal for gait analysis due to their impact on gait dynamics and their role in balance and movement [30,31,32]. In addition, they can be comfortable and practical if suitable sensors are selected for each area.

Kim et al. [21] developed sarcopenia detection models using gait data from 10 elderly women with sarcopenia and 10 women without sarcopenia, identifying spatiotemporal and descriptive statistical parameters of the gait phases. For feature selection and dimensionality reduction, they used Shapley values along with the XGBoost algorithm. They then applied ML algorithms such as Support Vector Machine, Random Forest, and deep learning (DL) algorithms, using the selected features as inputs.

Kim et al. [22] used logistic regression, a more traditional algorithm, employing the forward variable selection method to determine the associations between three speed-based gait variables and the loss of muscle mass, strength, and function in older women.

In their work, Kim et al. [23] utilized inertial-sensor-based wearable devices to analyze gait data for detecting osteopenia and sarcopenia in daily life. Gait signals were divided into seven phases, and descriptive statistical parameters were extracted for each phase. Algorithms including XGBoost, Random Forest, Support Vector Machine, and DL models were employed for classification. XGBoost achieved the highest accuracy for osteopenia, while Random Forest obtained the highest accuracy for sarcopenia. The authors used explainable AI techniques such as Shapley values to detect the importance and contribution of the parameters. Their findings highlight the high accuracy and significance of gait parameters for managing musculoskeletal diseases.

To predict sarcopenia in elderly women, Ko et al. [25] used IMU data collected during the Timed Up-and-Go test and 6-min walk test. They extracted 132 features and applied the Kruskal–Wallis test for feature selection. ML models, including k-nearest neighbors (kNNs), Support Vector Machine, and the Naïve Bayes algorithm, were trained, with kNNs achieving the best performance using 40 selected features. This study demonstrated the potential for self-monitoring sarcopenia using wearable devices.

Zhou et al. [26] investigated digital biomarkers of sarcopenia in frail elderly individuals through dual-task gait assessment. The authors used multivariate logistic regression analysis. Their study found that gait speed and turn duration during dual-task walking were significant predictors of sarcopenia, with turn duration showing the highest predictive ability. Combining these parameters improved the AUC, supporting their utility as sensitive biomarkers for sarcopenia detection in frail populations.

Obuchi et al. [29] explored the use of AI-based gait analysis to detect early cognitive impairment in community-dwelling older adults. Sensors attached to the pelvis and left ankle recorded triaxial linear acceleration and angular velocity data as participants walked at a comfortable speed. This study underscored the efficacy of analyzing a single walking stride using DL models to distinguish between individuals with and without cognitive impairment.

In addition to the unimodal approach, another limitation is that practical application is often limited; many studies rely on instrumented tests like the Timed Up-and-Go test, which diverges significantly from the natural movements that people perform in daily life [25,27,28], or use up to six sensors positioned across the body, making the system obtrusive and uncomfortable to wear and impractical to use [26,27]. In this context, studies focusing exclusively on gait analysis with a few number of sensors offer greater real-world applicability, as this approach is less invasive and monitors routine activity performed daily.

It is also noteworthy that many of these studies include samples composed only of women [21,22,23,25], which may limit the generalizability of results to individuals of other sexes.

Recent studies have also developed gait-based algorithms using computer vision (CV) algorithms or CV combined with IMUs to detect sarcopenia [24,33,34]. Within CV systems, human pose estimation (HPE) is a widely used task that enables detecting and tracking body joints to capture a person’s pose through AI techniques. By analyzing sequences of images or videos, these algorithms can detect gait patterns and joint angles, which help assess the functional state of the musculoskeletal system.

Combining smart insole data with AI-based pose estimation, Kim et al. [24] classified control and sarcopenia groups using Random Forest, Support Vector Machine, and artificial neural network models. Their study demonstrated high accuracy using pose estimation variables, with further improvements observed when combining insole and pose estimation data. These strong results were achieved particularly with the Random Forest model. The results emphasized the potential of advanced digital biomarkers like hip and ankle variables in identifying sarcopenia.

Libraries such as BodyFlow [35] have been developed in the multimodal context. This open-source and modular library was primarily designed for human activity recognition (HAR) and is capable of processing data from various sources, including videos, images, webcams, and IMUs sensors. BodyFlow includes advanced algorithms for 2D and 3D pose estimation, allowing the selection of an appropriate HPE model. Thanks to its modular structure, it not only enables activity recognition but also makes it possible to apply HPE algorithms to other areas, such as detecting diseases through gait analysis.

These systems based on vision, when implemented at home, could provide an effective and convenient method for screening and monitoring diseases such as sarcopenia and CD, as individuals would not need to wear any sensors or devices. However, concerns about the invasion of privacy with video-recording systems raise questions about their feasibility in real-world applications.

### 1.2. Our Proposal

The study we present aims to address the challenge of disease detection through gait analysis, using two complementary technologies (sensor fusion). Specifically, the main aim of this study was to develop AI algorithms based on gait parameters assessed through sensors and CV to detect sarcopenia and CD. The research overcomes the limitations of previous studies that rely solely on one technology (unimodal systems) and focus on a single disease. Additionally, our study includes data from both sexes and takes practical applicability into account.

To address the objective of this study, a comprehensive methodology was implemented. The first phase involved data collection. Then, the collected data were analyzed and their quality was assessed, identifying areas for improvement. Next, the most appropriate AI techniques and models were applied for data preprocessing, ensuring their quality and format for subsequent modelling. This phase also included features’ generation and selection, which were used as predictors in the models. Finally, models for detecting sarcopenia and CD were developed and evaluated, optimizing their performance. The paper is organized accordingly in the following sections, where each of these phases is explained in detail.

## 2. Materials and Methods

### 2.1. Study Design and Recruitment

A cross-sectional case-control study using a convenience sampling method was conducted. Individuals aged over 60 years from adult daycare centers, as well as able-bodied individuals residing in Zaragoza and nearby areas, were invited to participate. All methods were performed in accordance with relevant guidelines and regulations. This study was approved by the Clinical Research Ethics Committee of Aragon (CEICA) (PI23/084), and all participants provided written informed consent.

### 2.2. Definition and Assessment of Sarcopenia

Sarcopenia was defined according to the operational criteria and cut-off points proposed by the European Working Group on Sarcopenia in Older People (EWGSOP) [36]. Muscle strength was assessed using a handgrip strength test with a dynamometer (TKK 5001, grip A, Takei, Niigata, Japan). Participants were in a standing position, keeping the arm of the tested side straight down with the elbow extended (not touching the rest of the body). The best value from two attempts with each hand was recorded as the maximal grip strength.

Bioelectrical impedance analysis (BIA) and a body composition analyzer (TANITA MC780MA, TanitaCorp, Tokyo, Japan) were used to measure body weight and estimate appendicular skeletal muscle mass (ASM) using the formula proposed by Sergi et al. [37]. Physical performance was assessed using the Timed Up-and-Go test (TUG). Participants started in a seated position on a chair, stood up, walked 3 m at a normal gait speed, turned 180°, walked back to the chair, and sat down. The test was performed once and timed. Sarcopenia was diagnosed if the maximal grip strength was <27 kg in men or <16 kg in women, in addition to an ASM/height^2^ (ASMI) of <7.0 kg/m^2^ in men or <5.5 kg/m^2^ in women [36]. Sarcopenia was considered as severe if, in addition to the previous criteria, the TUG was ≥20 s in both genders [36].

### 2.3. Definition and Assessment of Cognitive Decline

Cognitive function was measured using the Spanish version of the Mini-Mental State Examination (MMSE) [38]. The MMSE consists of 11 items and is widely used in clinical and research settings to assess cognitive function across different domains. The total score ranges from 0 to 30, with higher scores indicating better cognitive function. CD was defined as a score of ≤24 points in the MMSE [38].

### 2.4. Gait Pattern Assessment

Participants were instructed to start from a standing position and walk at their normal, self-selected speed in the middle of an 8-m corridor. The participants were required to walk the length of the corridor four times, turning at both the starting and ending points. To assess gait pattern, two different technologies were used simultaneously, specifically, IMUs and CV.

#### 2.4.1. Inertial Measurement Units

**Figure 1:**
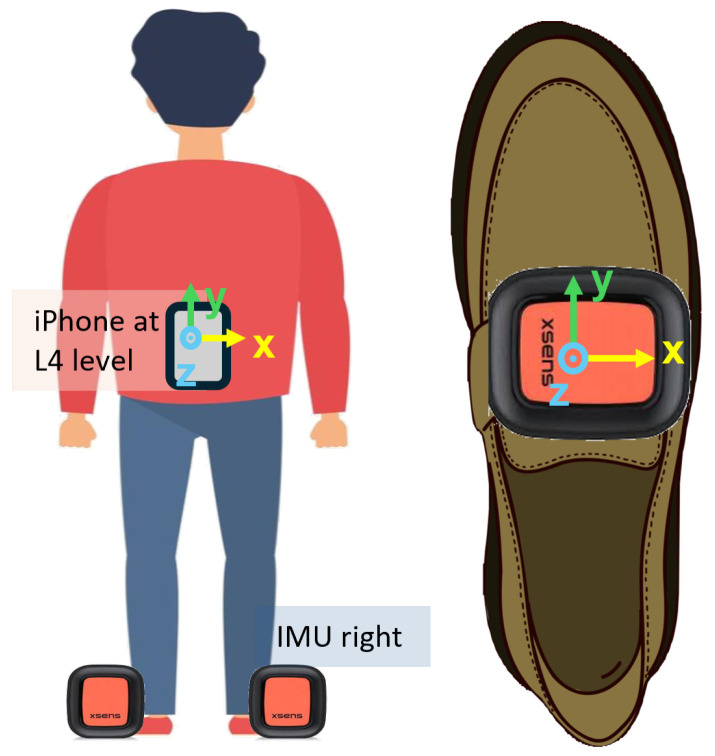
Sensor placement on participants.

As illustrated in Figure 1, during the test, participants were required to wear two IMUs (Xsens DOT, Movella Inc., Halifax, NS, Canada) attached to both feet and a smartphone (iPhone 15 Pro, Apple Inc., Cupertino, CA, USA, EEUU) attached to a belt and fixed at an L4 level. This combination of sensors was selected for its practicality and affordability and the opportunity to evaluate different types of devices in assessing gait dynamics. This approach aimed to balance the use of specialized sensors like Xsens for precision with widely available consumer-grade devices such as smartphones to ensure scalability and accessibility. To assess the gait pattern, 3-axis acceleration and angular velocity signals from both feet were recorded by the sensors at a sampling rate of 60 Hz. The IMUs’ raw data were directly recorded from the Xsens DOT sensors in CSV format for subsequent analysis. The smartphone also recorded 3-axis acceleration and angular velocity signals using Apple’s CoreMotion framework, which captured its raw inertial data, including the linear acceleration (userAcceleration) and rotation rate (rotationRate) along the x, y, and z axes.

#### 2.4.2. Computer Vision Equipment

During the test, digital images were also acquired. A GoPro HERO11 Black (GoPro, San Mateo, CA, USA, EEUU) with a GoPro Remote was used to capture video footage at a resolution of 3840×2160 pixels with a frame rate of 60 fps. The camera was positioned on a tripod at hip level, recording the test corridor from a lateral perspective to ensure the entire participant’s path could be captured. The lighting in each room was set as bright as possible, limited by the available resources in each testing location.

### 2.5. Data Analysis

Once the patients’ information was collected, a thorough analysis of the initial dataset was conducted. Data from the IMU sensors and images were evaluated to ensure their consistency and quality, considering factors such as accuracy, stability, sampling frequency, and image quality. The limitations detected guided the selection of the most appropriate preprocessing tools and led to the exclusion of patients whose conditions prevented the reliable extraction of gait patterns. This preliminary analysis also helped identify key areas in the time series and potential variables for summarizing gait patterns. All analyses were conducted using the Python programming language.

#### 2.5.1. Video Acquisition

Videos were recorded in various locations, leading to differences in lighting and background settings that could impact the accuracy of pose estimation models and, consequently, the extracted time series data for gait pattern analysis. Two primary challenges emerged: patients’ occlusion due to the presence of a person nearby to prevent potential falls during the walking test and inaccurate pose estimations, including the misidentification of left and right legs, caused by lighting conditions and dark clothing.

#### 2.5.2. Gait Pattern Analysis

**Figure 2:**
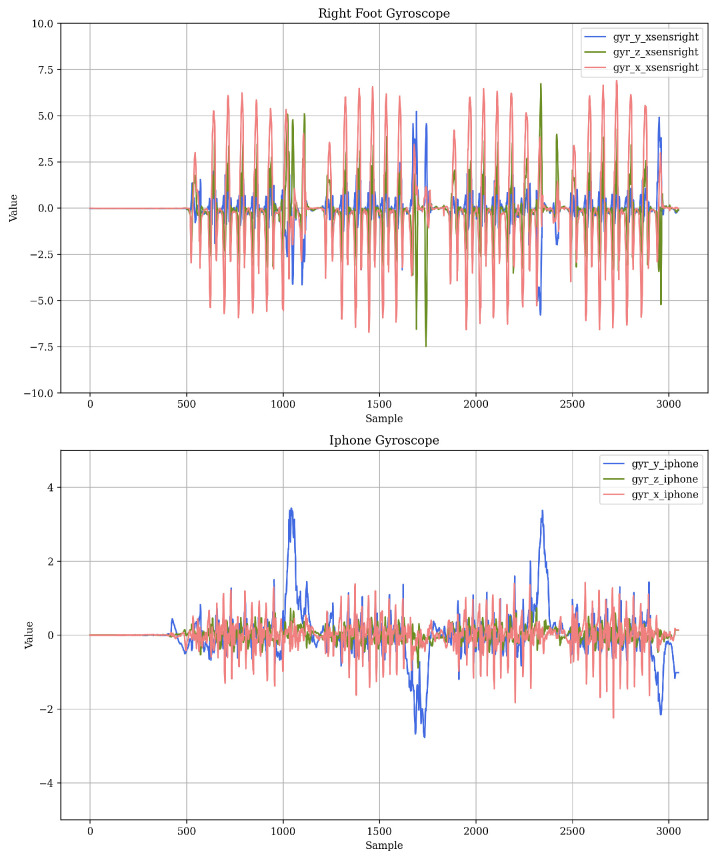
An example of values recorded by the gyroscope (rad/s) placed on the right foot (**top**) or on the back (**bottom**) during walking.

Figure 2 illustrates the time series data recorded for a subject by the gyroscope on the right foot (top) and the iPhone (bottom) during walking.

The upper figure presents the angular velocity values recorded by the sensor on the right foot, where four blocks corresponding to straight walking segments along the corridor can be distinguished. The *x*-axis, which shows the vertical rotation of the foot relative to the axis perpendicular to the ground per unit of time (Figure 1), is where the highest angular velocity values are observed, reflecting the most pronounced and representative movements of the gait.

In these blocks, repetitive patterns corresponding to the steps of the subject’s gait cycle can be observed, which include a local minimum, a global minimum, and a global maximum. The minima occur when the foot rotates toward the ground, while the maximum occurs when the foot moves in the opposite direction. These extremes are linked to the different phases of the step, from the foot’s contact with the ground to its forward motion, providing key information about the gait behavior.

At the end of each segment, the angular velocity values on the y and z axes of the foot show significant variations, in contrast to those recorded during straight walking. These changes correspond to the turns made by the subject at the end of the corridor, which involve lateral and dorsal rotations of the foot. Similarly, the iPhone gyroscope data on the *y*-axis (bottom figure) show peaks that reflect the back movements (rotations) occurring during the turns, with positive and negative values indicating the direction of the turns. Additionally, intervals where the angular velocity values approach zero are observed, particularly at the beginning of each block. This pattern suggests that the subject is at rest during these moments before initiating movement.

#### 2.5.3. Study Objective

The aim of this study was to develop models to detect sarcopenia or CD based on the analysis of participants’ walking patterns. The data collected included not only straight walking segments but also moments of turning and pauses at the beginning and end of walks, which are common situations in real measurement environments. The analysis focused on the behavior of continuous straight walking.

After the preliminary analysis of the data, the second walk was chosen as the main source of information for modelling for two reasons. First, some participants, especially the elderly or those with CD, were only able to complete two walks instead of the four requested. Additionally, the second walk was considered more stable and representative of their walking ability, as by that point they were more familiar with the environment.

#### 2.5.4. Final Study Population

A total of four subjects were excluded from this study. Two of them were removed because their information was not recorded at the same frequency during the walking activity. Additionally, two other patients required assistance from individuals holding them on both sides, which resulted in excessive occlusion in the images, preventing accurate pose estimation. The final study sample included 42 participants (57.14% female) with a median age of 70.5 years. In the overall sample, 7 participants had sarcopenia (including 3 with severe sarcopenia) and 17 had CD, according to the metrics and criteria used.

Data distribution between groups was summarized using medians and interquartile ranges for continuous variables and frequencies for categorical variables. The Mann-Whitney and chi-squared tests were applied as appropriate to assess group differences. A significance level of 0.05 (*p* < 0.05) was used.

### 2.6. Data Preprocessing

Once the data were analyzed, key aspects to consider were identified, and study objectives were defined, the next phase was data preprocessing. In this stage, techniques were applied to prepare and clean the study data, ensuring their quality and suitability for subsequent analysis.

The first step in data preprocessing focused on isolating stable walking periods (object of study), identifying moments when participants walked continuously without interruptions. The PELT [39] and dynamic programming algorithms were used to detect significant changes in the structure of the time series, such as variations in the mean or variance, facilitating the identification of alterations in the subject’s activity. In particular, to identify turns, the time series of the angular velocity recorded by the iPhone’s gyroscope (placed on the back) was analyzed. These algorithms accurately identified the start and end points of the turns, enabling the selection of the continuous walking period from the second walk for analysis. Finally, the algorithm was applied to the filtered time series to eliminate moments when the subject was immobile, such as pauses at the beginning or end of the walk, using the information recorded by the gyroscope located on the foot. This entire procedure ensured that the analyzed data exclusively represented a continuous and straight walk, without interruptions or turns.

Regarding the video data, the first step in preprocessing focused on video editing to address the challenges discussed in Section 2.5.1 (patient occlusion and inconsistent lighting). Subject overlap led to confusion in the model, as it would mistake the subject for their helper. To correct this, the target subject was segmented from the recorded frames using YOLOv8-seg [40], a state-of-the-art object detection algorithm, ensuring that the target subject was accurately separated from other people in the frame. People-tracking was performed with BoT-SORT [41], a multi-object tracking algorithm that enhances the basic SORT tracker by integrating re-identification, ensuring the consistent tracking of the target subject over time. Interfering individuals were then replaced by the median frame from the recording, maintaining only the background and effectively isolating the target subject.

Lighting conditions impacted leg detail visibility, especially when subjects wore dark clothing, which led to misidentifications between left and right legs, as already noted by other studies [42]. To resolve this, CLAHE adaptive histogram equalization from OpenCV [43,44] was applied to the segmented subject mask, with a clip limit of 10 and a tile grid size of 20×20 pixels. Specifically targeting the value channel in the HSV color scale enhanced brightness and contrast in darker areas, improving local detail and delineating the separation between the legs.

After preprocessing the video data, the next step involved estimating the subject’s pose and extracting the numerical time series data. To achieve this, the preprocessed video was analyzed using Bodyflow [35]. The MediaPipe module [45] was chosen due to its inclusion of foot keypoints essential for gait analysis. It provided 3D pose keypoints for the hip, knee, ankle, heel, and foot index, among others, with unnormalized coordinates that were then scaled with subject height and foot size.

**Figure 3:**
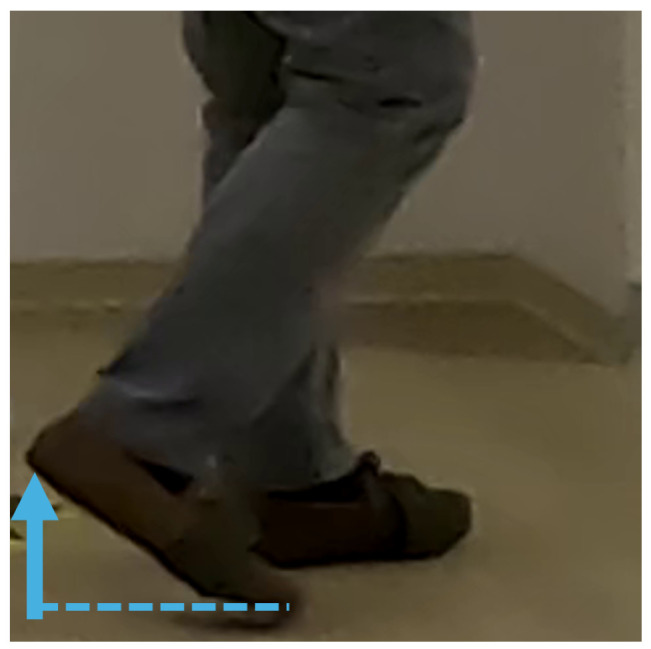
Processed foot vision signal: heel-to-toe height.

**Figure 4:**
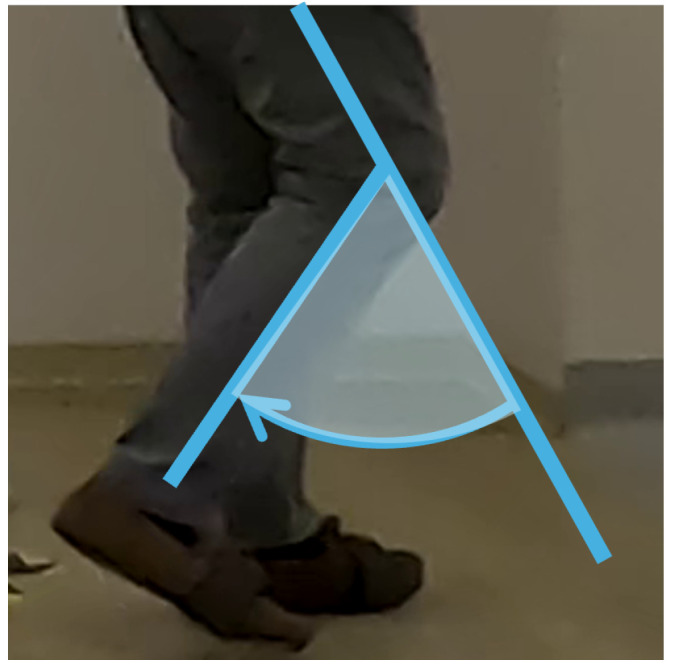
Processed knee vision signal: knee flexion angle.

**Figure 5:**
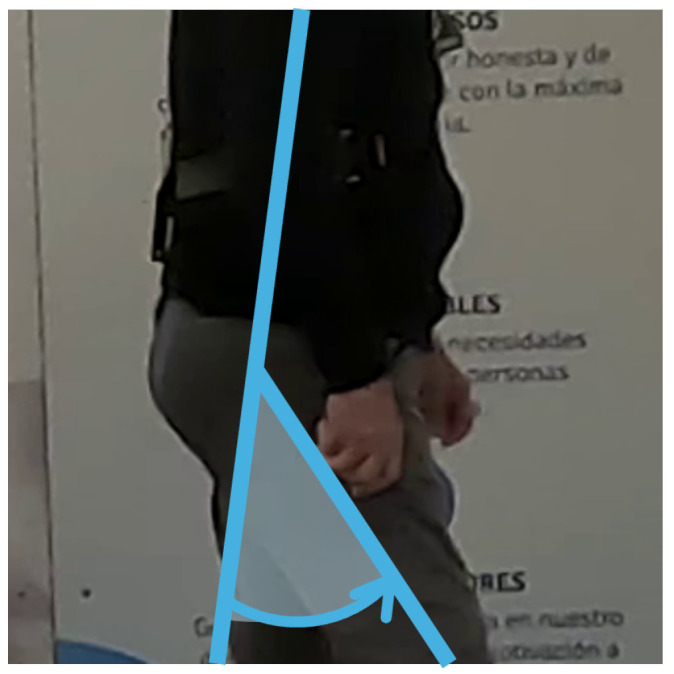
Processed hip vision signal: hip flexion angle.

To enable a more detailed gait analysis, flexion angles were extracted (deg), providing precise information for assessment. Consequently, body joints were projected to the subject’s sagittal plane, corresponding to the lateral view, from which the knee and hip flexion angles for both legs, as well as the heel-to-foot index height (metres) as a metric for the foot angle, were derived. The heel height-over-foot index was taken as positive, reaching its maximum at the initial swing (Figure 3). The knee flexion was defined as the angle between the femur and tibia, with a maximum during the swing phase (Figure 4). The hip angle, measured between the torso and femur, reached its maximum when the leg was brought forward (Figure 5). Unlike the IMU sensor data, these metrics represented angles rather than angular velocities and accelerations.

### 2.7. Feature Engineering

After analyzing, filtering, and preprocessing the time series data, the next step in the data preprocessing phase was feature engineering, which involved generating variables from the processed data. These variables helped summarize the walking behavior, which facilitated inferences in the final model. In the present study, we differentiated the variables based on their origin: on one hand, those obtained from the gyroscope and accelerometer (IMUs) sensors; and on the other hand, those derived from vision techniques, coming from camera recordings. This section presents the feature engineering process followed for each source of information. The user’s speed in the walk through the corridor was calculated based on the time taken and included as a variable in both the IMU sensor and vision-based analyses.

#### 2.7.1. IMU Sensors

Regarding the information obtained from the IMU sensors, various approaches were explored to generate the variables, including the detection of signal extremes, area calculations, and the identification of gait phases. Table 1 displays the 15 variables generated and included in the analysis, considering information from both the right foot IMU sensor and the iPhone, along with their corresponding descriptions.

As discussed in the previous data analysis phase (Section 2.5), the extremes of the foot gyroscope signal (*x*-axis) could serve as key indicators of gait behavior and followed a specific pattern. To generate useful variables, these extremes—local minima, global minima, and global maxima—were identified, corresponding to specific phases in the gait cycle. A simple moving average filter was applied to smooth the signal, which helped reduce the variability and noise present in the original data. This approach ensured that the extreme values more accurately represented the overall trends in the signal.

Specifically, the global minimum value of the signal in each step corresponded to the moment when the angular velocity reached its lowest value, which generally occurred when the foot rotated downward during plantar flexion. The global maximum value reflected the maximum angular velocity during forward movement or upward foot rotation, associated with the dorsiflexion phase. On the other hand, the local minimum referred to the change in rotation when the foot transitioned from heel contact to being fully supported on the ground. Based on these identified extremes, three variables were created: *foot_max_xsens*, *foot_local_min_xsens*, and *foot_global_min_xsens*. These variables captured the median values of the detected extremes during the gait, providing a summarized representation that robustly reflected the subject’s typical gait behavior. The number of steps (*steps*) was determined by counting the pairs of consecutive maxima, based on the repetitive pattern of each step (local minimum–global minimum–global maximum).

Additionally, derived variables were created to provide more information about gait dynamics. Specifically, variables were calculated to measure the signal’s amplitude, defined as the difference between the maximum and minimum values, which allows for assessing the overall range of motion during the gait cycle. The total area of the smoothed signal was also calculated to analyze gait dynamics. This was performed by summing the areas above and below the median of the signal and normalizing the result based on the amount of data to facilitate comparisons between subjects. The analysis was applied to the foot gyroscope signal across three axes, as well as to the *y*-axis acceleration (which measured forward movement) and iPhone gyroscope data. This total area analysis could help identify mobility deficits, as a smaller area may have indicated less rotation per unit of time, suggesting less dynamic and agile movement.

Finally, the variable *median_stance* was calculated, representing the median value of the percentage of time the individual spent in the stance phase during each step. In each gait cycle, the stance phase was defined as the period between two zero-crossings of the signal: the first occurred when the signal transitioned from the global maximum to the local minimum (at heel strike, when foot rotation stopped), and the second happened when the signal moved from the global minimum to the global maximum (at initial swing, when plantar flexion rotation stopped).

#### 2.7.2. Vision

**Figure 6:**
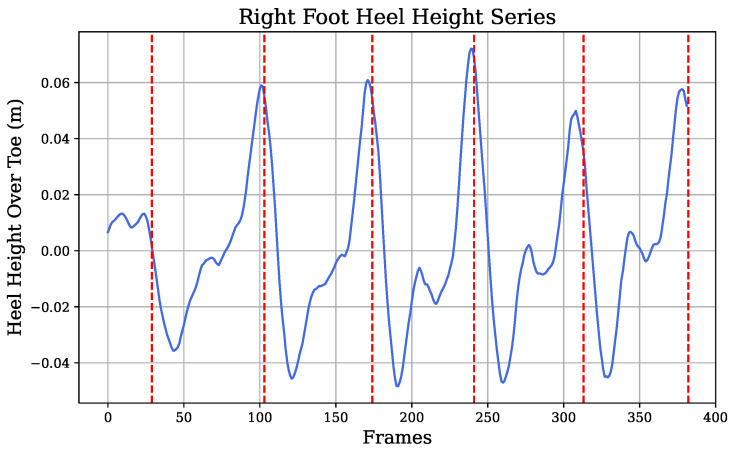
Heel height-over-toe signal.

**Figure 7:**
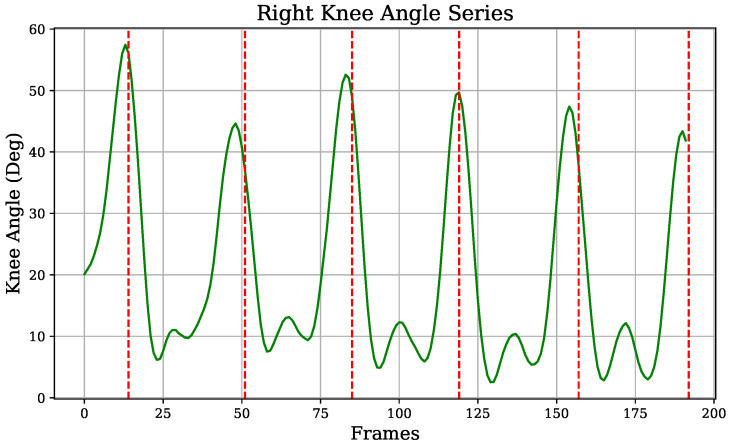
Knee flexion angle signal.

**Figure 8:**
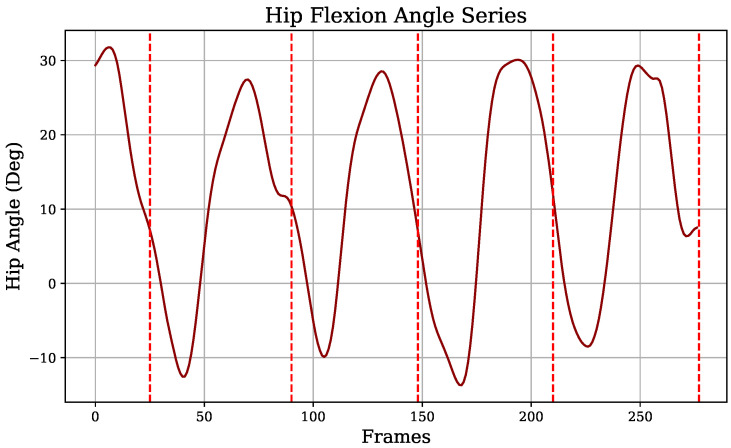
Hip flexion angle signal.

Concerning the vision-based information, as previously mentioned (Section 2.6), angle signals from the knee and hip flexion were extracted, in addition to the heel height-over-toe time series, from pose estimation. Each of these signals exhibited a characteristic gait pattern, with steps separated by red vertical lines in Figure 6, Figure 7, and Figure 8, respectively. Similar to the IMU sensors’ data, approaches identifying signal extremes and defining gait phases were applied to generate variables from vision-extracted signals.

Table 2 displays the 16 variables included in the analysis, derived from the pose estimation of the right foot, knee, and hip, along with their corresponding descriptions. To reduce noise, a simple convolution with an 11-element window was applied. Additionally, a detrending process was applied when needed to address signal issues caused by perspective distortions in the pose estimation.

In each step, extremes within the gait cycle were identified, generating variables such as *foot_max_vision*, *foot_min_vision*, *foot_max_amplitude_vision*, *foot_mean_amplitude_vision*, and *foot_median_amplitude_vision* from the heel-to-index height of the right-foot signal, as shown in Figure 6. Specifically, *foot_max_vision* and *foot_min_vision* represent the median of all maxima and minima within each step, respectively, providing robust measurements. For example, *foot_max_vision* captured peak plantar flexion in the initial swing phase, indicating the height that the heel was lifted to above the toe index, as illustrated in Figure 3. Amplitude variables, calculated as the difference between maxima and minima within a step, were summarized using maximum, mean, and median values across all steps to capture various approaches in the data.

Knee flexion angles series were similarly analyzed to identify their extremes, generating the variables *knee_min_vision*, *knee_max_vision*, *knee_max_amplitude_vision*, and *knee_median_amplitude_vision*, based on the maximum and absolute minimum values within each step, as shown in Figure 7. Specifically, knee_max_vision reflects the degree of knee flexion as the leg was brought forward during the swing phase, as seen in Figure 4.

Similarly, hip angles were captured, generating the variables *hip_min_vision*, *hip_max_vision*, *hip_max_amplitude_vision*, and *hip_median_amplitude_vision*, representing the maximum and minimum hip flexion across all steps (Figure 8). In particular, *hip_max_vision* indicates the forward extent of the leading leg during heel strike relative to the torso, as shown in Figure 5.

Finally, a posture-related metric, *hip_mean_angle_vision*, was derived as the average of the hip flexion angle signal, which reflects the overall torso inclination during the walking test. A higher average hip flexion angle suggests a more inclined torso posture. Additionally, *median_stance_vision* was calculated to represent the percentage of the gait cycle spent from the heel strike to toe-off, with the median taken across steps to provide a robust estimate.

Once the variables were defined and generated, and prior to starting the modelling phase, variables with a correlation higher than 0.9 were removed from each group of information sources (IMU sensors’ and vision-based information), as this indicated very similar relationships. This process helped reduce the dimensionality of the problem and prevent information redundancy.

### 2.8. Data Modelling

The main objective of this study was to develop predictive models for sarcopenia and CD using data from IMU sensors and vision. Six distinct models were created: three for sarcopenia (one based solely on IMU sensor data, another solely on vision data, and one combining both sources) and three for CD, following the same structure. This approach enabled assessing whether the combination of these data sources improved the model performance or whether a single source could provide accurate predictions on its own.

To address this, based on the generated and selected variables (Section 2.7), XGBoost was chosen for its proven efficacy in the field of machine learning. To determine the optimal model configuration, a hyperparameter search was conducted using the Optuna framework [46], which enables a dynamic search space through the Tree-structured Parzen Estimator (TPE) algorithm [47]. Five-fold cross-validation was used to select the best model, establishing the threshold that maximized the average F1-score, thereby prioritizing a balance between precision and sensitivity.

Once the best XGBoost model was identified, SHAP (SHapley Additive exPlanations) values [48] were calculated to interpret predictions and assess the importance of variables. This methodology assigns contributions to each variable, facilitating informed decision-making in clinical contexts and enhancing model explainability and transparency.

The information from the SHAP values also guided variable selection. Additional models were built incrementally—first using the three most relevant variables, then the four most relevant, and so forth—to assess if fewer variables could sustain, reduce, or even enhance model performance. For models that combined vision and sensor data, a two-step process was applied: (1) an initial model was constructed with selected variables from both data sources, identified through SHAP analysis, and (2) SHAP analysis was reapplied to refine the model, select the most suitable one, and identify the most relevant variables.

For each study object (sarcopenia or CD), the best-performing model was selected from those using sensor data, vision, or their combination. To benchmark this model against alternative algorithms, the optimal XGBoost models were compared, and additional models were developed using logistic regression (LR), Random Forest (RF), Support Vector Machine (SVM), and multi-layer perceptrons (MLP) with the selected variables. The model with the highest average F1-score across folds was chosen as the final model, along with its selected variables.

## 3. Results

Table 3 summarizes the distribution of the characteristics of the final study population, divided into two groups: sarcopenia versus non-sarcopenia and CD versus non-CD.

The median age was higher in the disease population (sarcopenia or CD), although a significant difference was only observed between the CD and non-CD groups. Patients with CD were shorter and weighed less. No significant differences in sex distribution were observed between the case and control groups (healthy individuals). As shown, participants with sarcopenia or CD also presented a lower ASM and reduced handgrip strength.

### 3.1. Feature Engineering

Table 4 shows the distribution of the IMU sensor-derived variables, comparing the two groups: sarcopenia versus non-sarcopenia and CD versus non-CD. This univariate analysis highlights significant differences in the distribution of each variable, providing insight into user behavior within each group.

Patients with sarcopenia and CD took more steps and walked at a significantly lower speed compared to the control groups. The control population exhibited higher maximum values for angular velocity. A similar trend was observed in the angular velocity during foot rotation toward the ground. These results were also reflected in the signal amplitude measurements, which were significantly lower in the disease group, indicating a reduced range of angular velocity. The variables measuring the total signal area showed significantly larger areas in the control population, suggesting a more dynamic gait pattern and a greater range of motion. Regarding the percentage of time spent in the stance phase, no significant differences were found between the sarcopenia and non-sarcopenia groups. However, in the CD group, the percentage was slightly higher.

Similarly, Table 5 illustrates the distribution of variables derived from vision-based techniques.

It was observed that the absolute angles of the foot in the disease-affected group were lower compared to the control group, indicating a reduced range of motion in the affected populations. While significant differences were found in these foot-related variables, no notable variations were observed in the knee- and hip-related variables. This suggests that foot dynamics may play a more significant and distinguishing role than hip and knee dynamics in detecting walking behavior. However, it is important to emphasize that while these analyses offer a preliminary understanding of the informative variables among the groups, they rely on a univariate approach that does not consider interactions between different variables. A multivariate approach should be considered to gain a deeper and more comprehensive understanding of the diseases under study.

### 3.2. Data Modelling

As mentioned in Section 2.7, before building the models, variables with a correlation greater than 0.9 were removed.

In the analysis of the IMU sensor-based variables, the following variables were removed due to high correlations with others: *foot_amplitude_xsens*, *foot_local_amplitude_xsens*, *area_sum_gyr_x_xsens*, and *area_sum_acc_y_xsens*. As a result, the set of sensor-derived variables was reduced to 11.

Similarly, for the vision-based data, the variables *foot_max_amplitude_vision*, *foot_mean_amplitude_vision*, *foot_median_amplitude_vision*, *knee_median_amplitude_vision*, *knee_max_amplitude_vision*, and *hip_max_amplitude_vision* were also removed due to exceeding a correlation of 0.9. As a result, the set of variables derived from vision data was reduced to 10.

Using the previous variables as inputs, the XGBoost algorithm was initially used to develop the models for detecting sarcopenia and CD. Table A1 presents the explored hyperparameter search range, as well as the optimal configuration achieved for each model.

#### 3.2.1. Sarcopenia

Table 6 presents the results of the discriminative capacity for the sarcopenia model, analyzing the model with only (IMU) sensor variables, vision variables, and the combination of both at the optimal cutoff point. The discriminative capacity was assessed using metrics such as the F1-score, sensitivity, specificity, precision, the negative predictive value (NPV), and accuracy, all derived from the average values obtained through cross-validation.

The results indicate that the model based solely on the sensors (11 variables) performed slightly worse in terms of the F1-score compared to the model based on vision data (10 variables), although the two were quite similar.

**Figure A1:**
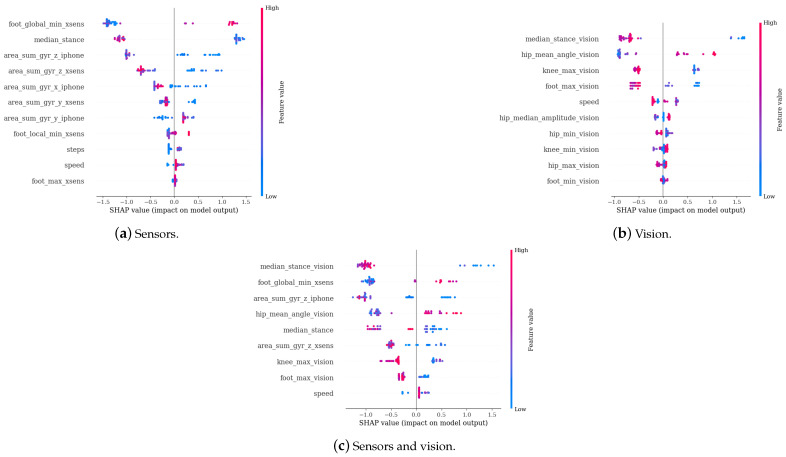
Comparative SHAP results for sarcopenia with all variables.

Figure A1 presents the SHAP plot corresponding to the models in Table 6, illustrating the global explainability and displaying the variables ranked by importance.

When evaluating the performance of the models using the most important variables iteratively according to SHAP, it was found that the sensor-based model achieved its best performance with only the four most important variables, while the vision-based model performed better with five variables. When combining both types of information, the best performance was obtained with four variables.

Table 7 presents the performance results of these three models, showing that the average F1-score was significantly higher when using selected variables compared to models that considered all initial variables (Table 6), highlighting the benefits of variable selection and minimizing potential noise. The model incorporating information from both sources achieved the highest F1-score, approximately 0.75, with a 100% sensitivity and around a 65% precision. This indicates that by combining information from both sources and applying variable selection, the model improved its predictive performance.

**Figure 9:**
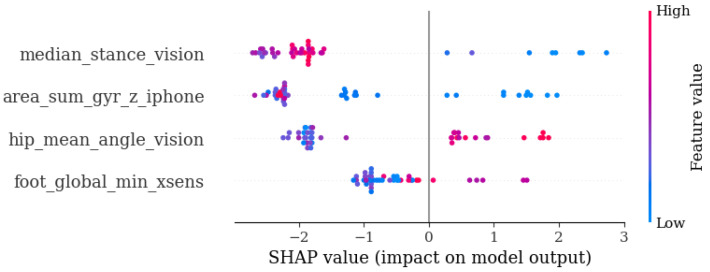
Sarcopenia final model with top 4 variables for sensors and vision.

Figure 9 displays the SHAP plot for the best sarcopenia detection model (based on both sensors and vision, Table 7), illustrating the overall interpretability of the model. The *x*-axis represents the Shapley value, while the *y*-axis displays the variables, organized from highest to lowest importance. The further the variables are from the vertical line, the greater their contribution is. Positive Shapley values indicate a risk of disease (sarcopenia), while negative values suggest the opposite. Additionally, the color of the variables represents their values, with blue indicating lower values and red indicating higher values.

Figure 9 shows that the variable measuring the percentage of the stance phase of the step captured through vision, along with the variable quantifying the total area of the gyroscope signal on the iPhone’s *z*-axis, were the most influential in the model. Additionally, the model included variables assessing the angles of the hip and foot, covering different characteristics and regions of the information. It is observed that a larger area of the gyroscope signal was associated with a lower risk of sarcopenia. A greater range of motion in this *z*-axis may have been linked to more movement in the hip (lower rigidity). The graph also shows that a lower hip angle (with a less inclined and more rigid torso) could act as a protective factor.

#### 3.2.2. Cognitive Decline

Table 8 presents the results of the discriminative capacity for the CD model, analyzing the model with only sensor variables, vision variables, and the combination of both at the optimal cutoff point. The results show that the models incorporating vision information had better performance.

**Figure A2:**
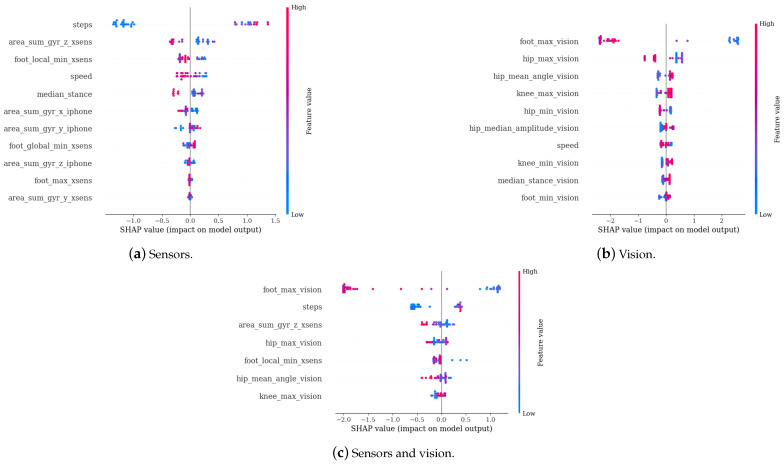
Comparative SHAP results for cognitive decline with all variables.

Figure A2 shows the SHAP plot for the models in Table 8, providing an overview of the global explainability and presenting the variables ranked by their importance.

Table 9 shows the results of the three final models with the best performance following variable selection based on the SHAP values. It is again evident that the models incorporating vision information outperformed the model relying solely on sensor data. Specifically, the model that used only four vision variables achieved the same F1-score as the one that also included sensor data.

**Figure 10:**
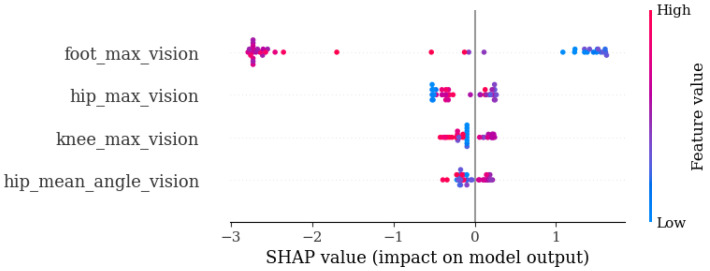
Final CD model with top 4 vision variables.

Figure 10 displays the explainability plot using the SHAP values for the best CD detection model (based on vision). This graph highlights that the variable measuring the maximum angle achieved by the foot were significantly more important than the others, contributing notably to the models’ decisions. Lower values (i.e., a smaller angle) were more strongly associated with a higher risk of CD, which could be linked to gait patterns characterized by increased foot dragging or reduced mobility.

#### 3.2.3. Comparison of Algorithms: Best Models

The best model for sarcopenia detection used four variables from the sensors and vision data, whereas the optimal model for CD detection relied exclusively on four vision-based variables. Using these sets of input variables, the performance of these models built with XGBoost was compared to other machine and deep learning algorithms. The aim was to determine which one provided the most robust and consistent performance (final model). The hyperparameter search space explored for each algorithm is detailed in Table A1.

Table 10 and Table 11 present the best results of different algorithms applied to sarcopenia and CD detection, respectively. In both cases, it is observed that the model with the highest F1-score was XGBoost, although for CD, the other algorithms also performed well with similar values. The model built with the XGBoost algorithm was selected as the best model due to its higher F1-score and high sensitivity (the percentage of patients with the condition correctly identified by the model).

## 4. Discussion and Conclusions

Sarcopenia and CD are highly prevalent diseases in elderly populations that can significantly impact healthcare systems and individuals’ quality of life. Detecting them in early stages is crucial, as these degenerative musculoskeletal and cognitive diseases can worsen over time. Early detection would allow for more personalized monitoring, improve the quality of life for those affected, and reduce associated costs. However, early detection remains a challenge due to the lack of screening tools that are both accessible and effective.

In this study, we presented a methodology for developing AI models capable of detecting the risk of sarcopenia or CD through the analysis of patients’ gait patterns. To achieve this, innovative technologies such as inertial sensors and CV were used. This practical approach aimed to improve the detection of these diseases and to serve as a support tool in clinical diagnostic screening systems.

The methodology involved analyzing the data obtained from both technologies and applying preprocessing techniques for the generation of AI models. This process included signal analysis, from which variables were generated that captured gait-related information from two different approaches, providing valuable information related to the user’s movement. On one hand, information from the angular velocity provided by the IMU sensors was used, while on the other, HPE techniques were applied to the recorded videos, allowing for the derivation of the patient’s foot, knee, and hip flexion angles. This multimodal approach enabled the analysis and integration of information from two different perspectives and body parts, offering complementary insights. Due to this diversity in the sources of information, the models were generated and evaluated both independently for each source and combined, in order to assess their predictive capability.

The results of this study show that both technologies, sensors and vision, are effective for detecting sarcopenia and CD, with the developed models achieving optimal performances. The models developed for CD detection demonstrated excellent performance. For sarcopenia, although the models’ performances were somewhat lower, the top-performing model still exceeded the performance of current screening systems used in healthcare settings [49].

When comparing the results of our study to previous studies that developed CD detection systems, we observe that the performance of our models for detecting CD is superior in most cases [14,50,51].

On the other hand, when comparing our study’s results to previous studies on sarcopenia detection, we observe that our models’ performance is comparable to those reported by other researchers, with accuracy rates ranging from 72.0% to 95.0% [13]. However, it is noteworthy that our study included participants of both sexes, thus avoiding the limitation of most studies that only include women [21,22,23,25], which enhances the external validity of our findings.

Specifically for sarcopenia detection, the model’s performance improved by combining both information sources and selecting a set of variables based on SHAP values. This suggests that adding more variables may have introduced noise or redundancy, reducing the effectiveness of the predictive features. In contrast, a proper selection of variables from both sources optimized the predictive performance in detecting this disease.

Regarding CD detection, the model that used vision-based information outperformed the one based solely on sensors. The explainability analysis with SHAP values also helped to understand the model’s decisions and the contribution of each variable to its predictive capacity. Concerning CD, the foot flexion angle was identified as an important predictor, where larger angles acted as a protective factor for the disease, suggesting less foot drag and greater mobility.

The performance difference between the sarcopenia and CD detection models developed in this study may have been partly due to the number of participants with each disease, with a much larger number presenting CD (n = 17) compared to sarcopenia (n = 7). Nevertheless, it is noteworthy that the models developed for detecting both sarcopenia and CD demonstrated high sensitivity, making them ideal as initial screening systems to detect these diseases in their early stages.

Our study tested two different technologies, both independently and, for the first time, in combination, paving the way for future research exploring new technologies for disease detection and their integration to achieve more robust screening systems. However, as other studies have noted, all screening systems based on current investigational technologies have advantages and limitations [4]. Various studies have demonstrated the potential of inertial sensors for movement analysis in real-world settings, as their portability and comfort rarely interfere with daily activities [52]. Nevertheless, wearing sensors on outdoor shoes and a belt while at home may be perceived as uncomfortable. On the other hand, vision-based systems have raised concerns within the research community regarding their acceptability, as some individuals might view them as intrusive to personal privacy [53,54]. However, vision-based systems offer distinct benefits, such as comfort, as they do not interfere with daily activities, require no wearable sensors, and do not need continuous management or manipulation by the person being monitored. This makes them potentially highly applicable, especially for older adults who may be less familiar with handling technology. Combining both systems, in addition to enhancing screening performance in some cases, would enable continuous and accurate monitoring both at home and outside, partially addressing any discomfort that other types of systems might present in certain situations.

The strengths of our study include the fusion of multiple technologies to develop algorithms for screening two highly prevalent diseases in older adults, as well as the high-quality data analysis conducted. In this regard, in addition to developing models with performances comparable to or exceeding those other studies, the methodology presented can also contribute to future research and implementations. It is important to highlight that the data preprocessing was a thorough process that used AI techniques to address real limitations and obtain accurate and high-quality information. Although the recordings were made in a controlled environment and supervised by professionals, the observed limitations could be similar to those that may arise in a real domestic setting. The recordings were made in three different locations, with varying lighting conditions, different clothing colors, and occlusions caused by the proximity of other people, who were present to prevent falls. Thus, the preprocessing methodology used could be effectively implemented in real systems in domestic contexts.

However, our study has some limitations. First, the cross-sectional analysis did not allow us to establish a causal, temporal link between the analyzed variables. Second, although the sample size of participants with sarcopenia was sufficient to develop the models, it was somewhat small and imbalanced compared to the group without sarcopenia. Third, it should be noted that some participants with sarcopenia also presented CD. However, it is challenging to find older adults with only one pathology, as multimorbidity is very common in this population.

To address these limitations, we propose the following lines of future research. First, we aim to validate our system in a longitudinal, uncontrolled recording environment, such as a home environment, where data from more patients can be collected over time. The creation of new models using additional historical patient data could also provide new insights, as it would allow us to observe their evolution over time.

In this context, we aim to generate and validate an end-to-end system. Integrating these detection models into libraries like BodyFlow [35] could create opportunities to develop continuous screening and monitoring systems capable of the ongoing assessment of individuals within their home environments. BodyFlow includes a module for detecting daily human activities, such as walking or falling. Thus, the system could recognize when a person is walking and apply detection algorithms based on their gait pattern. This would enable early alerts to healthcare professionals about potential gait abnormalities, which could signal the onset of diseases. The monitoring system would not replace the diagnostic process performed by healthcare professionals but rather serve as a diagnostic aid that could help relieve the healthcare system by enabling the earlier detection of diseases, thus allowing for more efficient and effective intervention.

In conclusion, this study presents a methodology focused on developing AI models to detect sarcopenia and CD through gait analysis, integrating multimodal information and achieving good performance. We believe that our research could have a significant impact on the early diagnosis of these diseases and the improvement of clinical interventions, enabling more precise and early detection. Furthermore, its application could be extended to other clinical and monitoring contexts, where gait analysis is essential for the identification of these pathologies.

## Figures and Tables

**Table 1:** Sensors’ variables.

Variable	Description
speed	Walking speed
steps	Number of steps
foot_max_xsens	Median value of detected maxima
foot_local_min_xsens	Median value of detected local minima
foot_global_min_xsens	Median value of detected global minima
foot_amplitude_xsens	Difference between foot_max_xsens and foot_global_min_xsens
foot_local_amplitude_xsens	Difference between foot_max_xsens and foot_local_min_xsens
area_sum_gyr_x_xsens	Relative total area of right foot gyroscope signal series on *x*-axis
area_sum_gyr_y_xsens	Relative total area of right foot gyroscope signal series on *y*-axis
area_sum_gyr_z_xsens	Relative total area of right foot gyroscope signal series on *z*-axis
area_sum_acc_y_xsens	Relative total area of right foot accelerometer signal series on *y*-axis
area_sum_gyr_x_iphone	Relative total area of iPhone gyroscope signal series on *x*-axis
area_sum_gyr_y_iphone	Relative total area of iPhone gyroscope signal series on *y*-axis
area_sum_gyr_z_iphone	Relative total area of iPhone gyroscope signal series on *z*-axis
median_stance	Median stance percentage from gait cycle

**Table 2:** CV variables.

Variable	Description
speed	Walking speed
foot_max_vision	Median value of maximum plantar flexion during initial swing phases
foot_min_vision	Median value of maximum dorsiflexion during heel strikes
foot_max_amplitude_vision	Maximum value of amplitudes between peak plantar flexion and dorsiflexion
foot_mean_amplitude_vision	Mean value of amplitudes between peak plantar flexion and dorsiflexion
foot_median_amplitude_vision	Median value of amplitudes between peak plantar flexion and dorsiflexion
knee_min_vision	Median value of minimum knee flexion during stance phases
knee_max_vision	Median value of maximum knee flexion as leg is brought forward during swing phases
knee_max_amplitude_vision	Maximum value of amplitudes between knee flexion peaks
knee_median_amplitude_vision	Median value of amplitudes between knee flexion peaks
hip_min_vision	Median value of minimum hip flexion when bringing supporting leg back during stance phase
hip_max_vision	Median value of maximum hip flexion when propelling leg forward during swing phase
hip_max_amplitude_vision	Maximum value of amplitudes between hip flexion peaks
hip_median_amplitude_vision	Median value of amplitudes between hip flexion peaks
hip_mean_angle_vision	Average hip flexion angle, reflecting overall back posture while walking
median_stance_vision	Median stance percentage from gait cycle

**Table 3:** Characteristics of study population.

Characteristic	Total	Non-Sarcopenia	Sarcopenia	Non-CD	CD
	**(N = 42)**	**(N = 35)**	**(N = 7; 16.7%)**	**(N = 25)**	**(N = 17; 40%)**
**Age (years)**	70.5 (66, 81)	69 (65.5, 75.5)	81 (77, 84.5)	67 (65, 70)	81 (73, 85)
*p*-value		0.07		**<0.001**	
**Sex (female)**	24 (57.14%)	20 (57.14%)	4 (57.14%)	14 (56%)	10 (58.82%)
*p*-value		1		1	
**Height (cm)**	159.5 (139.6, 175.5)	160.3 (144.2, 175.5)	155.7 (139.6, 174.8)	162.8 (147.9, 175.5)	154.8 (139.6, 174.8)
*p*-value		0.241		**0.005**	
**Weight (kg)**	68.2 (44.6, 103.7)	70.8 (49.5, 103.7)	55.1 (44.6, 74.4)	72.6 (49.5, 103.7)	61.7 (44.6, 90.9)
*p*-value		**0.002**		**0.006**	
**ASM (kg)**	15.9 (9.7, 25.3)	16.5 (12.6, 25.3)	13.0 (9.7, 18.3)	16.82 (12.92, 25.32)	14.59 (9.69, 21.95)
*p*-value		**0.012**		**0.037**	
**ASMI (kg/m^2^)**	6.19 (4.32, 8.36)	6.38 (5.17, 8.36)	5.25 (4.32, 6.15)	6.31 (5.17, 8.36)	6.03 (4.32, 7.98)
*p*-value		**0.001**		0.327	
**Handgrip (kg)**	25.2 (9.9, 52.2)	26.9 (11.3, 52.2)	16.3 (9.9, 26.4)	28.7 (25.0, 30.0)	19.9 (9.9, 39.4)
*p*-value		**0.008**		**0.004**	
**TUG (seconds)**	13.46 (5.07, 39.60)	11.69 (5.07, 26.36)	22.03 (10.01, 39.60)	10.82 (5.07, 11.70)	17.17 (5.73, 39.6)
*p*-value		0.120		0.214	

CD: cognitive decline; ASM: appendicular skeletal muscle mass; ASMI: ASM/height^2^; TUG: Timed Up-and-Go test; N: total number of patients; %: percentage of users with disease; median (Q1, Q3) for continuous variables; absolute (relative frequency) for categorical variables; *p*-values < 0.05 are highlighted in bold.

**Table 4:** Sensor variables’ distribution: sarcopenia vs. non-sarcopenia and CD vs. non-CD.

Variable	Non-Sarcopenia	Sarcopenia	Non-CD	CD
**speed**	1.02 (0.85, 1.18)	0.75 (0.51, 0.89)	1.11 (0.96, 1.19)	0.75 (0.54, 0.92)
*p*-value	**0.0217**		**<0.001**	
**steps**	6.00 (5.00, 8.00)	9.00 (7.50, 19.00)	6.00 (5.00, 6.00)	8.00 (8.00, 12.00)
*p*-value	**0.0181**		**<0.001**	
**foot_max_xsens**	5.99 (5.04, 6.48)	3.88 (3.52, 4.98)	6.04 (5.41, 6.70)	4.98 (3.76-5.68)
*p*-value	**0.0038**		**0.0041**	
**foot_local_min_xsens**	−4.40 (−4.91, −3.60)	−2.51 (−3.69, −1.54)	−4.40 (−4.78, −3.99)	−3.32 (−4.78, −1.63)
*p*-value	**0.0071**		0.1064	
**foot_global_min_xsens**	−8.08 (−9.10, −7.33)	−6.36 (−6.62, −4.49)	−8.45 (−9.05, −7.65)	−6.76 (−8.76, −5.17)
*p*-value	**0.0090**		0.0614	
**foot_amplitude_xsens**	13.96 (12.59, 15.46)	10.38 (8.13, 11.78)	14.46 (13.09, 15.5)	12.43 (8.87, 14.48)
*p*-value	**0.0033**		**0.0120**	
**foot_local_amplitude_xsens**	10.11 (9.2, 11.42)	6.13 (5.18, 9.24)	10.41 (9.31, 11.37)	9.16 (5.43, 10.11)
*p*-value	**0.0022**		**0.0241**	
**area_sum_gyr_x_xsens**	4.87 (4.13, 5.81)	2.8 (2.05, 3.94)	5.31 (4.43, 5.98)	3.58 (2.34, 4.38)
*p*-value	**0.0019**		**<0.001**	
**area_sum_gyr_y_xsens**	61.10 (52.06, 79.24)	46.65 (41.66, 60.57)	62.82 (51.98, 81.65)	56.32 (46.65, 65.83)
*p*-value	0.0576		0.1827	
**area_sum_gyr_z_xsens**	73.63 (59.10, 101.22)	57.53 (52.32, 58.24)	76.74 (65.87, 120.17)	57.53 (48.03, 69.38)
*p*-value	**0.0213**		**0.0025**	
**area_sum_acc_y_xsens**	0.87 (0.69, 0.97)	0.54 (0.44, 0.69)	0.88 (0.74, 0.97)	0.63 (0.45, 0.79)
*p*-value	**0.0487**		**0.0090**	
**area_sum_gyr_x_iphone**	19.37 (15.4, 29.53)	17.61 (16.73, 19.37)	23.91 (17.48, 29.63)	16.57 (14.1, 18.76)
*p*-value	0.5295		**0.0334**	
**area_sum_gyr_y_iphone**	29.02 (23.82, 39.67)	22.20 (20.50, 25.32)	29.87 (24.69, 40.49)	25.09 (22.20, 29.02)
*p*-value	**0.0313**		0.0689	
**area_sum_gyr_z_iphone**	18.22 (14.04, 24.13)	11.74 (9.86, 13.78)	18.31 (14.55, 24.34)	13.90 (11.28, 17.64)
*p*-value	**0.0029**		**0.0356**	
**median_stance**	69.14 (67.38, 71.78)	68.96 (68.29, 75.85)	68.80 (67.08, 69.49)	72.02 (68.96, 74.24)
*p*-value	0.4263		**0.0120**	

CD: cognitive decline; median (Q1, Q3); *p*-values < 0.05 are highlighted in bold.

**Table 5:** CV variables’ distribution: sarcopenia vs. non-sarcopenia and CD vs. non-CD.

Variable	Non-Sarcopenia	Sarcopenia	Non-CD	CD
**speed**	1.02 (0.85, 1.18)	0.75 (0.51, 0.89)	1.11 (0.96, 1.19)	0.75 (0.54, 0.92)
*p*-value	**0.0217**		**<0.001**	
**foot_max_vision**	0.04 (0.04, 0.05)	0.03 (0.02, 0.03)	0.05 (0.04, 0.06)	0.03 (0.02, 0.04)
*p*-value	**0.0081**		**<0.001**	
**foot_min_vision**	−0.03 (−0.05, −0.03)	−0.03 (−0.03, −0.02)	−0.04 (−0.05, −0.03)	−0.03 (−0.03, −0.02)
*p*-value	0.1141		**0.0027**	
**foot_max_amplitude_vision**	0.10 (0.09, 0.12)	0.07 (0.06, 0.08)	0.10 (0.10, 0.12)	0.07 (0.07, 0.09)
*p*-value	**0.0063**		**<0.001**	
**foot_mean_amplitude_vision**	0.08 (0.07, 0.09)	0.06 (0.04, 0.06)	0.09 (0.08, 0.10)	0.06 (0.05, 0.07)
*p*-value	**0.0174**		**<0.001**	
**foot_median_amplitude_vision**	0.08 (0.07, 0.10)	0.06 (0.04, 0.07)	0.09 (0.08, 0.10)	0.06 (0.05, 0.07)
*p*-value	**0.0141**		**<0.001**	
**knee_min_vision**	9.25 (4.74, 17.28)	10.00 (6.28, 13.14)	14.84 (4.11, 17.69)	9.25 (6.26, 10.95)
*p*-value	0.9211		0.9796	
**knee_max_vision**	40.34 (23.58, 54.76)	34.86 (27.21, 42.60)	44.72 (13.61, 60.79)	39.85 (37.96, 42.69)
*p*-value	0.3884		0.8778	
**knee_max_amplitude_vision**	44.77 (32.55, 51.27)	32.94 (29.74, 47.23)	43.19 (14.84, 52.73)	45.03 (35.52, 47.15)
*p*-value	0.6676		0.8778	
**knee_median_amplitude_vision**	34.21 (19.41, 42.94)	24.01 (20.97, 33.80)	33.97 (10.14, 44.38)	32.86 (25.48, 40.25)
*p*-value	0.5740		0.6263	
**hip_min_vision**	−2.82 (−7.18, 2.60)	1.14 (−2.64, 8.00)	−2.82 (−7.99, 2.79)	−2.18 (−3.35, 3.20)
*p*-value	0.1310		0.2705	
**hip_max_vision**	25.15 (19.09, 30.47)	24.56 (21.25, 29.32)	28.16 (16.30, 32.88)	24.56 (22.02, 26.46)
*p*-value	0.8950		0.7978	
**hip_max_amplitude_vision**	34.59 (25.21, 42.78)	31.07 (20.54, 33.44)	36.41 (21.98, 45.09)	31.89 (28.20, 34.60)
*p*-value	0.3187		0.5218	
**hip_median_amplitude_vision**	27.80 (18.85, 36.48)	24.45 (14.48, 29.09)	31.29 (14.89, 37.74)	27.14 (21.25, 28.38)
*p*-value	0.4868		0.5386	
**hip_mean_angle_vision**	11.33 (9.72, 14.95)	13.46 (12.59, 16.31)	11.12 (9.74, 14.99)	12.69 (10.46, 14.45)
*p*-value	0.1496		0.7585	
**median_stance_vision**	69.44 (68.14, 71.99)	62.54 (62.08, 67.29)	70.19 (68.31, 71.79)	68.58 (62.54, 69.44)
*p*-value	**0.0113**		**0.0485**	

CD: cognitive decline; median (Q1, Q3); *p*-values < 0.05 are highlighted in bold.

**Table 6:** Performance metrics for sarcopenia model. Different variable sets with total variables.

	F1-Score	Sensitivity	Specificity	Precision	NPV	Accuracy	Threshold
Sensors	0.527	0.700	0.886	0.5	0.95	0.856	0.3
Vision	0.560	0.800	0.771	0.483	0.938	0.764	0.1
Sensors and Vision	0.500	0.600	0.914	0.5	0.921	0.858	0.4

NPV: negative predictive value.

**Table 7:** Performance metrics for sarcopenia model. Different variable sets with selected variables.

	F1-Score	Sensitivity	Specificity	Precision	NPV	Accuracy	Threshold
Sensors	0.614	1	0.686	0.487	1	0.739	0.1
Vision	0.647	0.8	0.857	0.650	0.946	0.833	0.2
**Sensors and Vision**	0.748	1	0.829	0.647	1	0.858	0.1

NPV: negative predictive value; sensors (4): *foot_global_min_xsens*, *median_stance*, *area_sum_gyr_z_iphone*, *area_sum_gyr_z_xsens*; vision (5): *median_stance_vision*, *foot_max_vision*, *hip_mean_angle_vision*, *knee_max_vision*, *speed*; sensors and vision (4): *median_stance_vision*, *area_sum_gyr_z_iphone*, *hip_mean_angle_vision*, *foot_global_min_xsens*. Bold indicates the model with the best performance in terms of the F1-score.

**Table 8:** Performance metrics for CD model. Different variable sets with total variables.

	F1-Score	Sensitivity	Specificity	Precision	NPV	Accuracy	Threshold
Sensors	0.734	0.750	0.840	0.743	0.860	0.806	0.5
Vision	0.892	0.950	0.880	0.860	0.967	0.906	0.3
Sensors and Vision	0.914	0.950	0.920	0.900	0.967	0.928	0.8

NPV: negative predictive value.

**Table 9:** Performance metrics for CD model. Different variable sets with selected variables.

	F1-Score	Sensitivity	Specificity	Precision	NPV	Accuracy	Threshold
Sensors	0.787	0.817	0.840	0.777	0.887	0.831	0.7
**Vision**	0.914	0.950	0.920	0.900	0.967	0.928	0.8
**Sensors and Vision**	0.914	0.950	0.920	0.900	0.967	0.928	0.8

NPV: negative predictive value; sensors (3): *steps*, *area_sum_gyr_z_xsens*, *foot_min_local_xsens*; vision (4): *foot_max_vision*, *hip_max_vision*, *knee_max_vision*, *hip_mean_angle_vision*; sensors and vision (4): *foot_max_vision*, *steps*, *area_sum_gyr_z_xsens*, *hip_max_vision*. Bold indicates the model with the best performance in terms of the F1-score.

**Table 10:** Sarcopenia: sensors and vision.

	LR	RF	SVM	MLP	XG
**F1-Score**	0.605	0.633	0.389	0.514	**0.748**
**Sensitivity**	1.000	0.900	1.000	0.800	1.000
**Specificity**	0.657	0.771	0.257	0.686	0.829
**Precision**	0.480	0.607	0.255	0.420	0.647
**Accuracy**	0.714	0.783	0.383	0.714	0.833
**NPV**	1.000	0.975	1.000	0.967	1.000

Bold indicates the best in terms of the F1-score.

**Table 11:** Cognitive decline: vision.

	LR	RF	SVM	MLP	XG
**F1-Score**	0.864	0.893	0.903	0.914	**0.914**
**Sensitivity**	0.900	0.900	0.883	0.900	0.950
**Specificity**	0.880	0.920	0.960	0.960	0.920
**Precision**	0.850	0.900	0.950	0.950	0.900
**Accuracy**	0.883	0.908	0.928	0.931	0.928
**NPV**	0.927	0.927	0.933	0.933	0.967

Bold indicates the best in terms of the F1-score.

## Data Availability

The data are not public due to ethical reasons.

## Abbreviations

The following abbreviations are used in this manuscript:

AI	Artificial intelligence
ASM	Appendicular skeletal muscle mass
ASMI	ASM/height^2^
BIA	Bioelectrical impedance
CD	Cognitive decline
CV	Computer vision
DL	Deep learning
EWGSOP	European Working Group on Sarcopenia in Older People
HAR	Human activity recognition
HPE	Human pose estimation
IMU	Inertial measurement units
kNN	k-nearest neighbors
LR	Logistic regression
ML	Machine learning
MLP	Multi-layer perception
MMSE	Mini-Mental State Examination
RF	Random Forest
SHAP	SHapley Additive exPlanations
SVM	Support Vector Machine
TPE	Tree-structured Parzen Estimator
TUG	Timed Up-and-Go test
XG	XGBoost

## Appendix A. Results: Data Modelling

### Appendix A.1. Hyperparameters Explored

**Table A1:** The search space of the hyperparameters explored for each algorithm.

Model	Parameters	Search Space	Best Model: Sarcopenia	Best Model: Cognitive Decline
RF	Number of Estimators	[50, 500]	132	204
	Max. Depth	[2, 9]	3	7
	Min. Samples Split	[2, 10]	4	10
	Min. Samples Leaf	[1, 4]	3	2
	Max. Features	[None, sqrt, log2]	log2	None
SVM	Kernel	[rbf, sigmoid, linear]	sigmoid	rbf
	C	[0.001, 10]	0.248	0.072
	Gamma	[$10^{-5}$, 10]	$8.63\times 10^{-5}$	1.93
	Tol	[$10^{-6}$, 0.1]	$6.03\times 10^{-6}$	0.0167
MLP	Number of Layers	[1, 5]	1	1
	Units	[16, 256]	176	256
	Dropout Rate	[0.0, 0.5]	0.42	0.33
	Batch Size	[32, 64, 128]	32	32
	Epochs	[10, 50]	28	10
	Activation	[relu, tanh, linear]	tanh	relu
	Optimizer	[adam, sgd]	adam	adam
	Learning Rate	[$10^{-4}$, 0.1]	0.0056	0.069
	Beta First Moment	[0.8, 0.99]	0.958	0.932
	Beta Second Moment	[0.9, 0.9999]	0.983	0.986
	Epsilon	[$10^{-8}$, $10^{-4}$]	$1.53\times 10^{-5}$	$3.09\times 10^{-5}$
	Weight Decay	[$10^{-6}$, $10^{-2}$]	$3.92\times 10^{-3}$	$1.52\times 10^{-6}$
XG	Max. Depth	[2, 9]	8	6
	Learning Rate	[0.01, 0.2]	0.124	0.0188
	Number of Estimators	[50, 200]	156	141
	Subsample Size per Tree	[0.8, 1.0]	0.804	0.834
	Colsample per tree	[0.8, 1.0]	0.994	0.813
	Scale of Positive Weights	[1, 5]	5	5
	Gamma	[0, 0.1]	0.0212	0.0966

*Sgd:* stochastic gradient descent; *Adam:* adaptive moment estimation optimizer; *Max. Depth:* maximum depth of tree; *Min. Samples Split:* minimum number of samples required to split internal node; *Min. Samples Leaf:* minimum number of samples required at leaf node; *Max. Features:* fraction of features considered when splitting; *Units:* neurons per layer; *Dropout Rate:* fraction of units dropped to prevent overfitting; *Activation:* activation function for neurons; *Beta:* decay rate for first or second moment estimates in Adam; *Epsilon:* small constant to prevent division by zero in Adam; *Weight Decay:* L2 regularization coefficient in Adam; *Number of Estimators:* trees in ensemble methods; *Kernel:* kernel type in SVM; *C:* regularization parameter in SVM; *Gamma:* kernel coefficient in SVM or minimum loss reduction to split node in XGBoost; *Tol:* tolerance for stopping criterion in SVM; *Subsample Size per Tree:* fraction of data per tree in XGBoost; *Colsample per Tree:* fraction of features per tree in XGBoost; *Scale of Positive Weights:* balance between positive and negative classes.
